# Surface Enhanced Raman Spectroscopy of Planetary Materials Using Laser Deposited Plasmonic Gold Nanoparticles

**DOI:** 10.1021/acsomega.6c05378

**Published:** 2026-08-12

**Authors:** Atchutananda Surampudi, David Tuschel, Tilak Hewagama, Shahid Aslam, Dina Bower, Anil Aryal, Felix Jaetae Seo, Narasimha Prasad, Mool C. Gupta

**Affiliations:** † Charles L. Brown Department of Electrical and Computer Engineering, 744402 University of Virginia (UVA), Charlottesville, Virginia 22904, United States; ‡ LBT Scientific Consulting, Madison, Wisconsin 53714, United States; § 53523 NASA Goddard Space Flight Center, Greenbelt, Maryland 20771, United States; ∥ Laser and Plasma Technologies (LPT), LLC, Charlottesville, Virginia 22903, United States; ⊥ 3726 Hampton University, Hampton, Virginia 23668, United States; # 53524 NASA Langley Research Center, Hampton, Virginia 23681, United States

## Abstract

Detection of trace materials on planetary surfaces and changes to the surface composition or structural modifications is important for identifying contaminants and other near-surface changes that may contain valuable geological information. This work demonstrates a surface-enhanced raman spectroscopy (SERS) approach based on laser deposition of Au plasmonic nanoparticles onto several relevant mineral surfaces. A pulsed 1064 nm wavelength laser is used to ablate a thin gold film and deposit Au nanoparticles directly onto selected regions of an olivine mineral sample as the substrate, enabling localized in situ SERS probing. Thin surface layers of four examples of relevant planetary simulants, such as CSM-LHT, JSC-RN, OPRH4-W30, and NU-LHT-4M, which identify as lunar highland and Martian soil analogs, were deposited on olivine substrate to emulate surface-level deposits. After nanoparticle deposition on simulants, Raman features associated with these plagioclase-rich simulants became significantly more distinct, whereas conventional Raman measurements of the same thin layers produced only weak or incomplete signatures. The technique was also shown to detect a mixed surface layer containing JSC-RN simulant and TiO_2_ (anatase), demonstrating sensitivity to multiphase systems. The results indicate that laser-deposited plasmonic nanoparticles can enhance Raman detection of thin surface materials on rough mineral substrates with an effective enhancement on the order of ∼10^4^, offering a promising route for in situ planetary material analysis. This work demonstrates a path for in situ identification using SERS of planetary minerals with suppressed Raman signatures from space weather exposure. These results will advance planetary surface analysis by enabling localized in situ detection of thin surface deposits, alteration layers, and mixed mineral phases on rough substrates, with relevance to lunar and Martian exploration, astrobiology, geological sensing, and future instrument development for trace material detection in challenging environments. The same approach is relevant to geological surface analysis on Earth, where thin alteration layers, mineral coatings, and fragile surface deposits can be difficult to probe using conventional Raman methods, and the demonstrated method would be suitable for analyzing a variety of solid surfaces.

## Graphical abstract

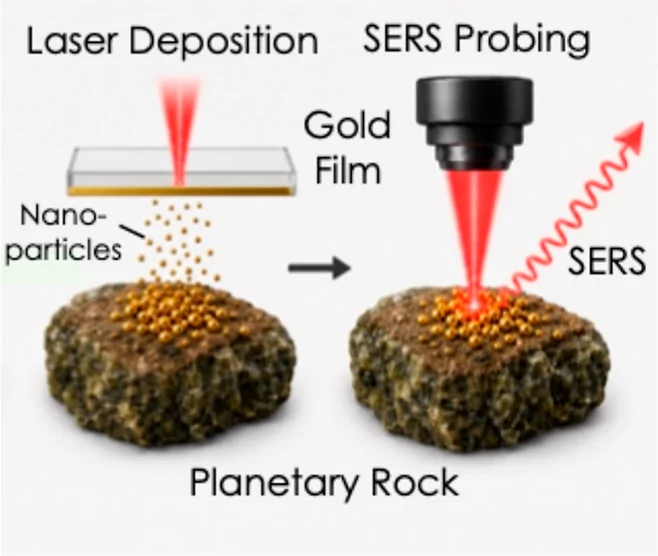

## 1 Introduction

Detecting trace components in surface materials or changes to surface properties from radiation exposure and extremes in temperatures is a central challenge in planetary science, astrobiology, and future exploration missions.^1^ Martian and lunar deposits are constantly modified by space weathering (micrometeorite impacts, cosmic radiation), oxidation, dust accumulation, and surface mixing over long time scales.^2^ As a result, the uppermost layers of rocks and regolith may contain thin surface coatings, alteration products, or sparse phases that preserve important compositional and environmental information. Raman spectroscopy is widely used for mineralogical analysis to provide information on chemical composition and biological signatures because it is nondestructive and chemically specific.^3,4^ But its intrinsically weak Raman scattering efficiency makes trace detection difficult, especially when the target material is present in trace amounts or as a thin layer on a substrate of mixed composition. Surface enhanced raman spectroscopy (SERS) has addressed this limitation by using localized plasmonic electromagnetic fields from noble-metal nanostructures to amplify Raman signals by many orders of magnitude.^5–7^ In the planetary context, SERS has shown promise for trace analysis of biologically relevant materials in sediments and Mars-analog soils.^8^

The technique of SERS works on the principle of plasmonic resonance. Plasmonic resonance is the collective oscillation of conduction electrons in a metal nanostructure driven by light, producing strong absorption, scattering, and localized electromagnetic near-fields.^9^ For conducting metallic nanoparticles such as Au and Ag, the resonance wavelength depends strongly on particle size, shape, spacing, and dielectric environment, so it can be tuned across the visible and near-infrared range. In SERS, this resonance is important because the local near-field can greatly amplify the Raman signal from material located very close to the nanoparticle surface.^10^ The strongest enhancement typically occurs when the excitation wavelength overlaps or lies close to the nanoparticle resonance, which is why matching the plasmonic absorption band to the Raman laser wavelength is a central design principle in SERS substrates.

For NASA-relevant planetary exploration, Raman and SERS methods are attractive because they can provide mineralogical and biomolecular information with minimal sample preparation. They are therefore relevant to the detection of alteration phases, hydrated minerals, carbonaceous materials, and potential biosignatures in planetary materials. In the context of the Moon, Mars, and other airless bodies, such techniques are particularly valuable because they can support the identification of trace chemical and structural information directly from rocks, soils, and surface coatings. Recent planetary instrumentation efforts have also reinforced the importance of surface-sensitive spectroscopic methods for mission concepts aimed at geological characterization and biosignature detection.^11–13^

Important material on planetary bodies is often concentrated in the uppermost few microns to nanometers of the surface,^14^ making surface sensitive methods attractive. Dust coatings, weathering products, irradiation-induced changes, and thin alteration films can all carry information about surface history, environmental exposure, and local chemistry. Because these thin surface layers lie well within the Raman probe penetration depth, near-surface signal enhancement is required to isolate their signatures from the underlying substrate.

Previous studies using noble metal particles dispersed using a liquid medium have demonstrated the utility of SERS for the detection of trace organics in Mars-like soils and sedimentary materials, including subppm detection in analog environments and immobilized analytes.^15–17^ Bowden et al.^15^ showed that SERS can provide large signal enhancements for molecular detection in rocks and sediments, while Tang et al.^16^ demonstrated fluorescence-free 1064 nm SERS for trace organics in Martian soil analogs. Whitaker et al.^17^ further showed that direct placement of a liquid containing a dispersion of organic and minerals on SERS-active substrate observed Raman signal enhancement. Collectively, these works establish SERS as a powerful diagnostic tool in planetary sensing, especially where analyte abundance is low, and conventional Raman spectroscopy is not sensitive enough.

Regarding SERS methods, a related and rapidly developing area is the fabrication of SERS-active substrates by laser-based techniques. Laser-based fabrication has emerged as a versatile route for preparing SERS-active nanostructures because it avoids multistep chemical synthesis and can produce clean metallic surfaces with tunable plasmonic response.^18–20^ In techniques such as pulsed laser deposition (PLD), laser ablation in liquids, laser-induced aggregation, and direct laser writing, the laser parameters can be adjusted to control nanoparticle size, shape, density, and spatial arrangement, thereby tailoring the Raman enhancement behavior of the resulting surface. Particularly, the reviews of PLD-based SERS substrates further note that this control on particle size and shape is also influenced by performing the experiment under inert or ambient environments.^19,20^ Experimental studies have shown that nanosecond laser treatment of thin gold films can generate Au nanoparticles with high SERS performance,^21,22^ while laser-deposited Ag nanoparticle patterns have demonstrated that enhancement depends strongly on nanoparticle density and spatial distribution.^23,24^ More recently, direct laser-induced writing has been used to create gold nanosphere SERS patterns with active areas defined on demand.^25^ These studies collectively show that laser-assisted nanoparticle generation is a viable and highly adaptable strategy for making SERS substrates.

Despite progress in laser-based methods, most prior work has focused on producing SERS substrates that are later used to detect separate analytes, typically liquids, molecular films, or functionalized surfaces, rather than on direct in situ probing of thin surface layers on rough solid surfaces such as minerals. That distinction is important for planetary applications, where the target is not a liquid analyte on a flat support but a heterogeneous solid surface with sparse near-surface materials. Another alternative to using SERS substrates is liquid colloidal dispersions of prefabricated nanoparticles, which are drop-casted onto a surface for SERS enhanced detection. However, for NASA mission-related applications, the use of liquid for metal nanoparticle coating is not an option. For space-relevant applications, liquid handling is undesirable and, in low-temperature, vacuum, and microgravity environments, may be impractical. A dry, localized, on-demand approach, such as that of a laser, that generates and deposits nanoparticles directly on the region of interest, is therefore especially mission enabling. In essence, the laser-SERS literature has largely concentrated on the fabrication of active substrates for later chemical sensing, rather than on the direct interrogation of the sample surface itself. In most cases, the laser-generated nanoparticles are transferred to a support SERS substrate and then used to detect liquid analytes, deposited molecules, or functionalized films. For planetary materials analysis, however, the sensing problem is fundamentally different. The target is regolith or a rough solid surface, often with sparse surface deposits or weathered layers, and the measurement must be performed without disturbing the sample. This important need motivates the present study.

This work addresses this need by using laser deposition of Au plasmonic nanoparticles directly onto a relevant planetary surface simulant to enable in situ SERS probing of thin surface layers. A 1064 nm wavelength pulsed laser is used to ablate a sputtered gold film on a transparent glass substrate, generating nanoparticles that are transferred to selected regions of an olivine mineral sample. Olivine is chosen as a representative base mineral substrate because it is relevant to both lunar and Martian materials.^26,27^ Thin surface layers of lunar and Martian simulants are prepared on top of the olivine substrate to emulate surface deposits, and the deposited nanoparticles are then used to interrogate these layers without disturbing the surrounding surface. This approach allows control over the deposition area, nanoparticle density, and effective particle size through the laser scan conditions, while avoiding the limitations of liquid colloids and prefabricated SERS substrates.

The novelty of this work lies in the direct, in situ use of laser-generated plasmonic nanoparticles to probe thin surface materials on planetary-relevant mineral substrates, rather than fabricating conventional SERS substrates for separate analyte detection. To the best of the knowledge, such an approach for enhancing Raman detection of thin surface layers or surface alterations on planetary materials has not been previously reported. The results demonstrate the ability to reveal Raman signatures from surface-level thin film simulants and mixed-phase deposits that are otherwise weak or undetectable by conventional Raman techniques, while preserving the underlying bulk mineral signal. Although the present work is motivated by planetary exploration, the same surface-sensitive sensing strategy may also be useful for Earth-based geological materials, including weathered rocks, sediments, and mineral coatings, and a variety of solid surfaces. In the sections that follow, the manuscript first describes the laser-deposition process and the sample preparation methods, then presents the Raman-SERS measurement results, and finally discusses the implications and impact of the approach for planetary material sensing.

## 2 Experimental Details

### 2.1 The Laser Deposition of Plasmonic Gold Nanoparticles

The concept of operation for laser deposition of nanoparticles is shown in Figure 1a. A laser beam (wavelength 1064 nm, pulse duration 30 ns, maximum pulse energy 1 mJ) is focused onto a thin metallic film of gold coated on a donor substrate (borosilicate glass, thickness ∼1 mm, 2 × 2 mm^2^) that is optically transparent to the laser wavelength. A 1064 nm wavelength is chosen as it was available in the laboratory and is a fundamental mode of laser operation, allowing higher laser efficiency in the delivery of pulse energy compared to other harmonic wavelengths.

**Figure 1:**
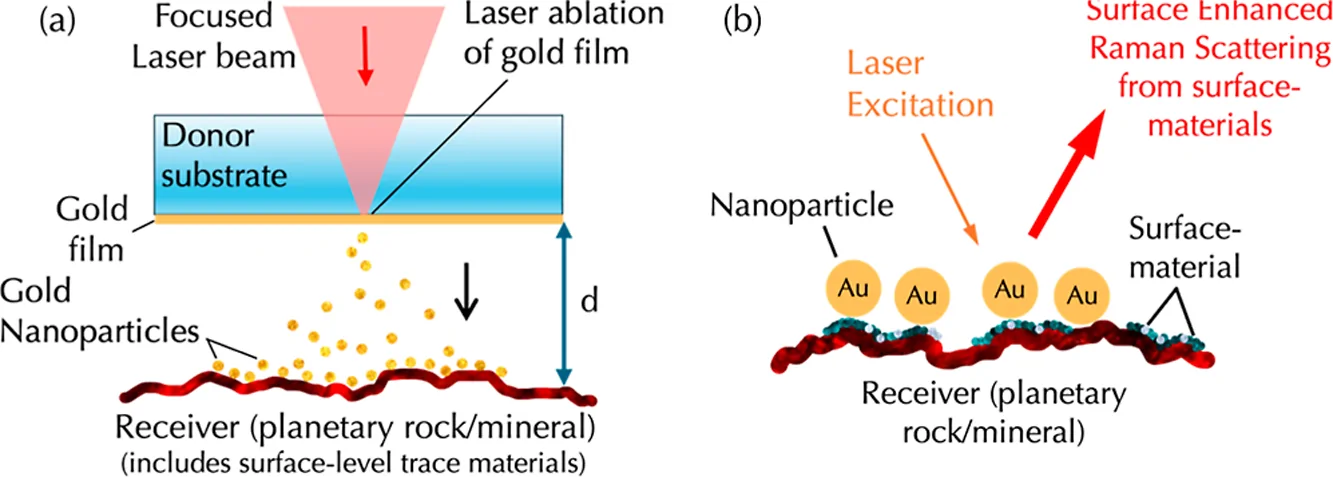
(a) The schematic diagram of the laser transfer method, (b) SERS probing from the receiver.

To prepare the donor substrate, the substrate surface is cleaned with a solution of isopropyl alcohol (IPA), sonicated under deionized (DI) water for 2 min, and dried on a hot stage at ∼100 °C. This clean substrate is then sputter coated with a film of gold to a thickness of 50 nm ± 3 nm. The sputtering is performed using a Cressington sputter coater with a gold target (Sigma-Aldrich), under argon gas at a pressure of 0.05 mbar, current of 20 mA, and for a duration of 80 s. This results in a continuous film of gold across the donor substrate.

At a sufficient laser fluence (∼5 J/cm^2^), the 50 nm gold film undergoes ablation, resulting in the formation of nanoparticles. For each ablation event, the laser beam is focused to a spot size of 50 μm on the gold film and scanned over an area of 200 × 200 μm^2^, at a speed of 1000 mm/s, with a laser operating at 20 kHz of pulse repetition rate. This ensures only one pulse per spot of ablation over each 50 μm spot, without overlap between adjacent spots. The ablation experiments are performed under ambient air, which highlights the simplicity of the experiment.

The receiver material is placed at a distance d of ∼5 mm to ensure a spatially uniform spread of the nanoparticles on the receiver. The receiver has surface-level trace materials, which are to be detected. Previous studies report similar distances and show the dependence of nanoparticle size distribution on the donor-receiver distance.^28,29^ With a single laser-pulse ablation event, the particles spread over a ∼1 mm^2^ area on the receiver. The nanoparticles are driven toward the surface of the planetary material by the momentum of the expanding laser generated ablation plume.

### 2.2 Measurement of Raman Scattering

As depicted in Figure 1b, the prepared surface is then probed for Raman measurements with a laser excitation wavelength that overlaps the plasmonic resonance exhibited by the nanoparticles. This results in the Raman signal from the surface-level material being enhanced, which allows their sensitive detection. The surface enhanced Raman signals accompany the Raman scattering from the bulk of the planetary material, depending upon the absorption depth of the laser wavelength into the material. The Raman measurement parameters (over the Renishaw InVia instrument) are as follows. For all the measurements, a laser wavelength of 785 nm is used at 50% laser power (maximum laser power of 120 mW) of the Raman instrument, and the measurements are made under backscattering geometry using a 50× objective. At the spectrometer-side, a grating of 1800 l/mm is used, which allows a resolution of 0.5 cm^–1^, with measurements made in the range of 200 cm^–1^–900 cm^–1^, over an integration time of 10 s, averaged over 2 scans. The Raman signals from the materials used in this study lie in the specified spectral range, and narrowing the range allows the detection with high spectral resolution.

### 2.3 Image Analysis

The SEM and EDS images are obtained using the FEI Quanta 650 instrument, under the concentric backscatter (CBS) detector, under the A + B + C mode with high vacuum (HV) driven at 15 kV. The Hirox RH 8800 high-resolution optical microscope was used to obtain the surface images of the receiver samples. Further statistical analysis of the SEM images to determine the nanoparticle size distribution (mean diameter and standard deviation) was performed using the ImageJ software.

### 2.4 Sample Preparation

The samples are prepared as follows. An olivine mineral sample (sourced from Ward’s, U.S.A.) is used as the base planetary material substrate. Olivine is chosen as it is a typically available mineral component in lunar and Martian rocks. There are four planetary simulants (sourced from NASA Goddard Space Flight Center) available in the powdered form, which are applied as a thin layer on top of the olivine sample, emulating the surface-level material/surface alterations on the olivine sample. The four simulants consist mainly of the plagioclase mineral^30,31^ and are described in Table 1.

**Table 1:** Planetary Simulants Used in This Study

	simulant	target/class	composition
1	CSM-LHT-1	lunar highlands	70 wt % plagioclase +30 wt % basaltic cinders (glass bearing basalt fragments/agglutinates, pyroxenes)
2	JSC-RN	martian soil	50 wt % plagioclase, 15 wt % pyroxene, 35 wt % additives (iron oxides, volcanic glass, feldspathoids)
3	OPRH4-W30	lunar highlands	90 wt % plagioclase, 10 wt % basaltic cinders
4	NU-LHT-4M	lunar highlands	65 wt % plagioclase, 35 wt % agglutinates

The thin layer of each powdered simulant is prepared as follows. A solution of 90:10 vol % DI water: isopropyl alcohol (IPA) was first prepared, and then, in a 50:50 volume % ratio, a given powder simulant was dispersed in the water-IPA solution. The 10% IPA in the DI water solution prevents the coagulation of the simulant powder in the dispersion. The top surface-layer of the prepared dispersion was extracted, and gently applied on the surface of olivine mineral, while it was being heated at a spatially uniform temperature of ∼100 °C on a hot stage. At the evaporation temperature of DI water, the grains of the simulant powder in each case settle over the surface of the olivine mineral sample.

Apart from the powdered simulants, a mixture of JSC-RN and TiO_2_ (anatase) powder (10:90 volume % ratio) was prepared to demonstrate the sensitive capability of the laser deposition technique to detect different components of a 2-phase system as well. The same dispersion-and-evaporation technique was used to prepare a thin layer of the mixture on the olivine sample, as shown in Figure 2.

**Figure 2:**
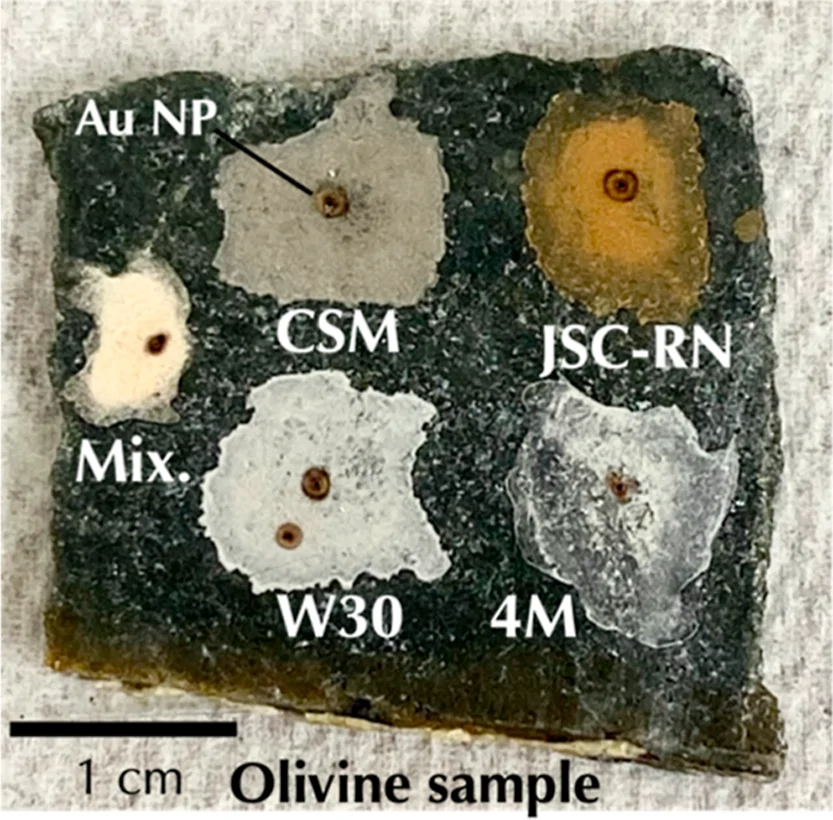
Olivine mineral sample with thin layers of different planetary simulants (CSM, JSC-RN, W30, 4 M) and the mixture (mix. of JSC-RN and TiO_2_ anatase) sample are applied on its surface. The Au nanoparticles (NPs) are selectively deposited in specific regions on each of the simulant areas and the mixture area.

Gold nanoparticles are deposited using the laser technique on each of the simulant thin-layer and the mixture. This capability to selectively deposit and contain the nanoparticles within a certain area for in situ SERS probing of the considered area, without affecting the surrounding regions on the thin-layer, highlights the novelty of the laser technique compared to other conventional methods.

## 3 Experimental Results

### 3.1 Laser Deposition

The SEM image of the thin gold film on the donor substrate after laser ablation is shown in Figure 3, depicting both the regions of gold film area and the laser ablated area exposing the glass substrate. It could be observed that under laser ablation, the gold film is removed and exposing the underneath glass substrate.

**Figure 3:**
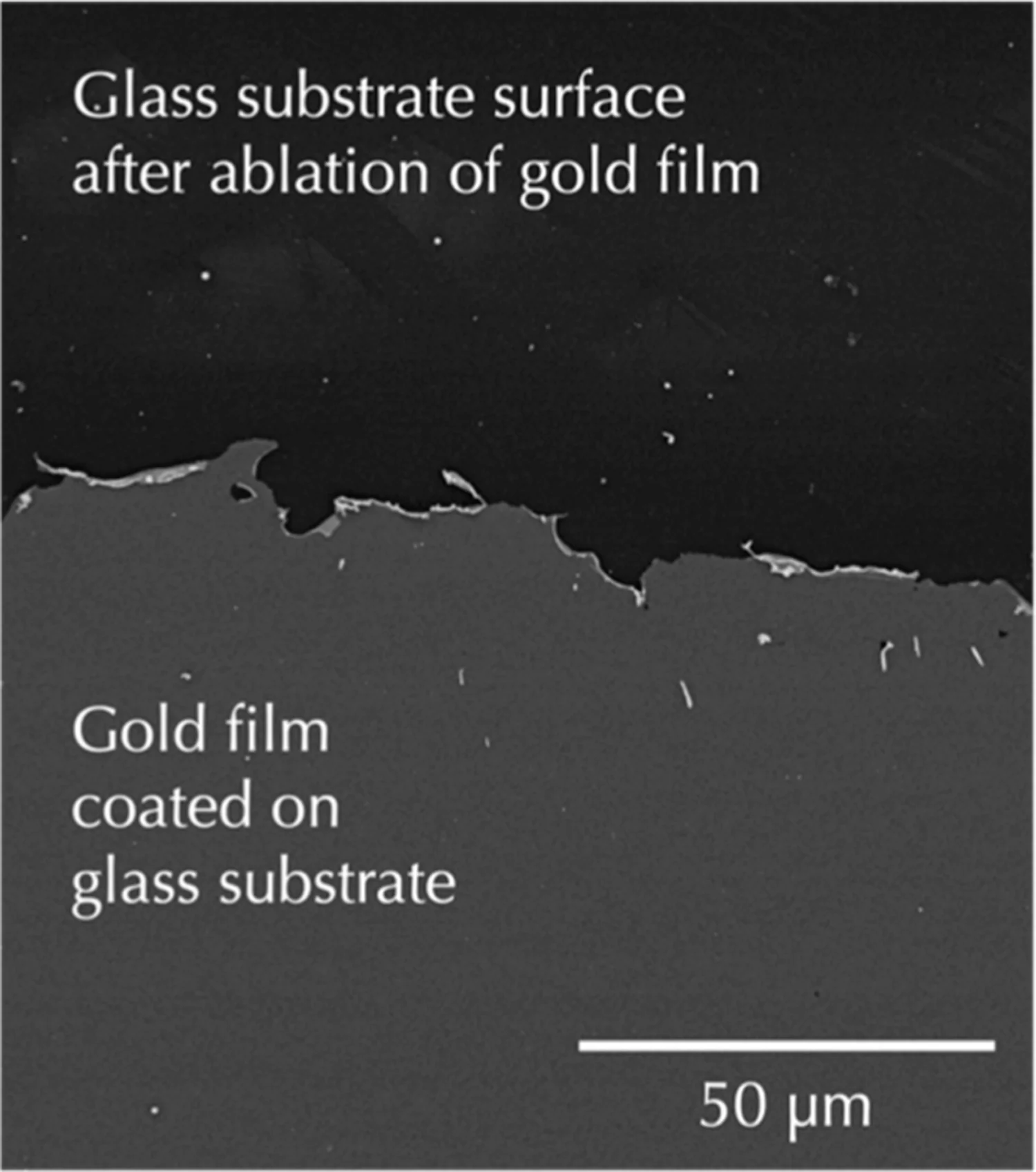
Donor substrate. It shows the gold film and the glass substrate after laser ablation. After ablation, the coated gold film is removed, exposing the glass surface.

The SEM image of the nanoparticles formed on an olivine substrate, as a receiver surface, is shown in Figure 4a. In this case, the laser peak power of 30% of the maximum. Was used for the deposition. The EDS measurements in Figure 4b reveal the elemental composition of olivine, which is an Fe–Mg silicate, along with the presence of Au (∼21.6 wt %) from the deposited gold nanoparticles. To achieve a sufficient spatial density of nanoparticles, the deposition is performed over 50 times at the same location at the receiver from different regions of the donor.

**Figure 4:**
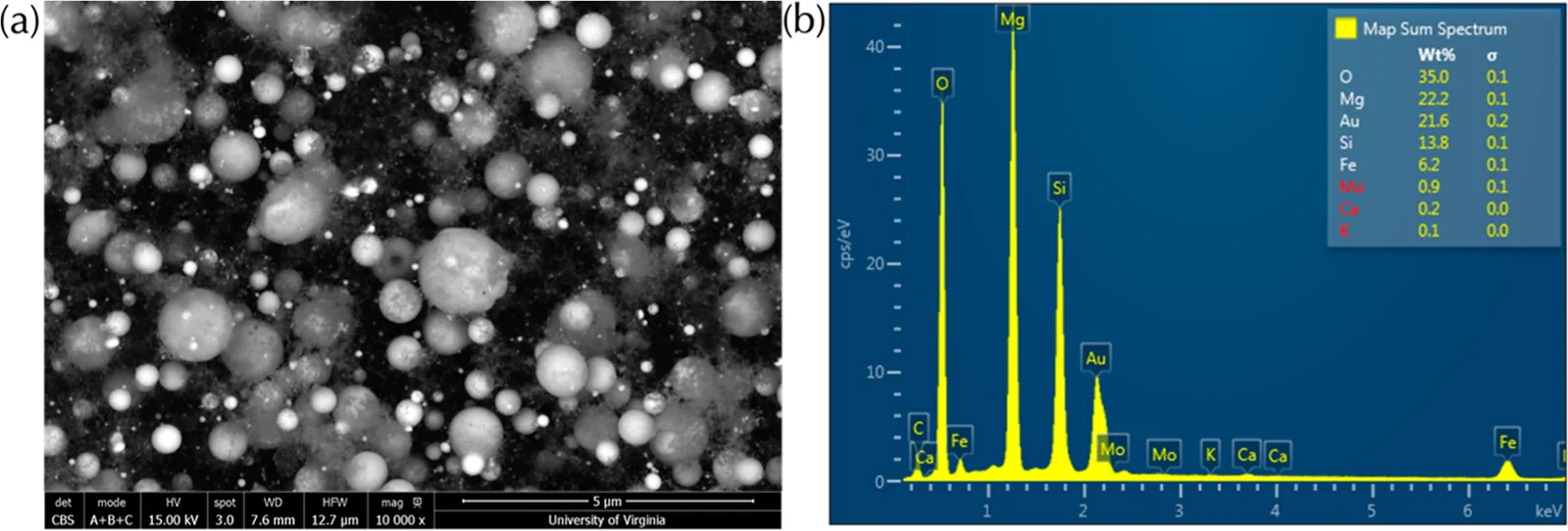
(a) SEM image of gold nanoparticles, (b) EDS measurements.

The statistical size distribution of the deposited nanoparticles is obtained as shown in Figure 5a. A mean size of 208.960 ± 107 nm is observed. When the laser power % is increased to 40% and 50%, the mean value drops to respectively 192.770 ± 103 nm and 162.579 ± 88 nm, as shown in Figure 5b,c, respectively. This reveals the flexibility of the laser-based approach to prepare surfaces with a control over the mean size of the nanoparticles, which in-turn controls the plasmonic resonance wavelengths.

**Figure 5:**
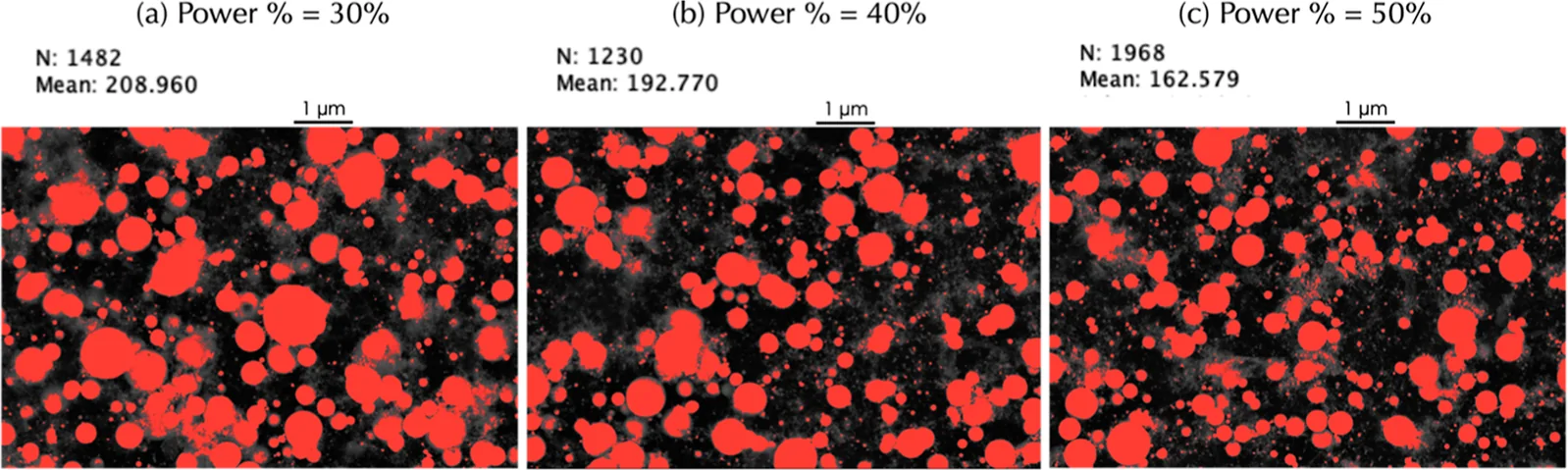
Size distribution of deposited nanoparticles at different power %: (a) 30%, (b) 40%, (c) 50% of peak laser power of 7.5 kW. N represents the number of particles in the image being analyzed by the ImageJ software.

Plasmonic resonance measurements are described next using Figure 6. To measure the plasmonic resonance wavelength, the gold nanoparticles are deposited (using 30% of the peak laser power) over a separate glass substrate as the receiver, as shown in the inset of Figure 6b. A 30% laser power is used because it is intended to obtain particles of larger mean diameters, allowing the plasmonic resonance in the longer wavelengths. The scan area on the donor substrate in this example is ∼2 × 2 mm^2^, which allows a deposition over a wider area over the receiver (which also shows the flexibility of the technique in controlling the particle deposition area. The absorption measurements were made using Cary UV–vis spectrometer for both the sample (Au nanoparticle deposited on the glass substrate) and the reference (bare glass substrate). The measurements are shown in Figure 6a. The normalized absorption for the sample is plotted in Figure 6b. A plasmonic resonance peak at ∼720 nm is observed. Corresponding to this plasmonic resonance, the Renishaw Raman instrument operating at a laser wavelength of 785 nm was available, which allowed the Raman and SERS measurements in the spectral band indicated in Figure 6b. Although not at the peak of resonance, this allowed sensitive detection of Raman peaks, as discussed in the following Section 3.2. A better wavelength for Raman measurements would nonetheless be ∼700–720 nm.

**Figure 6:**
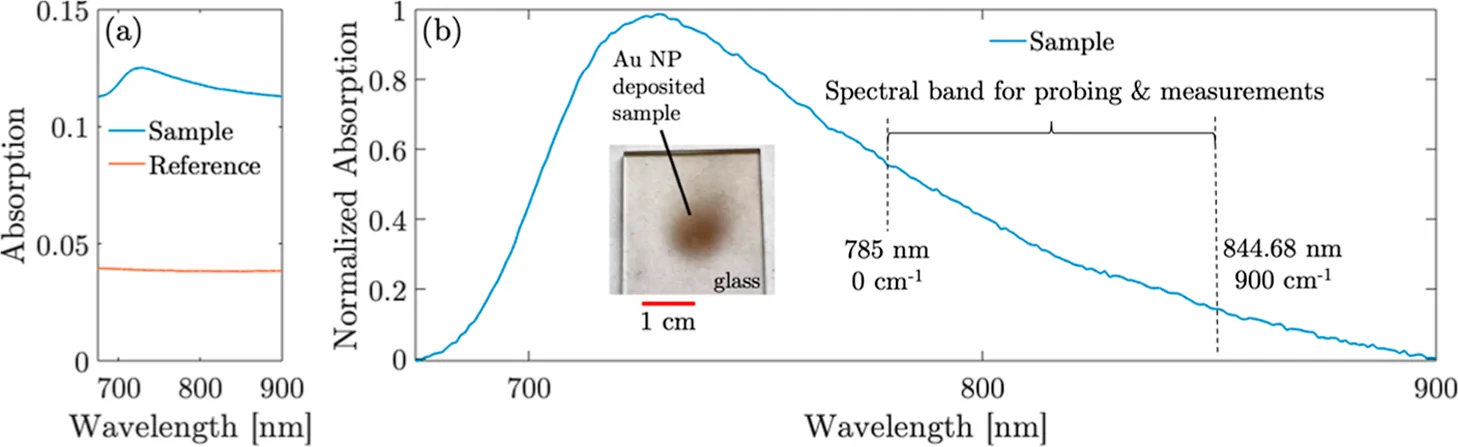
Absorption spectrum. (a) Absorption spectrum of Au nanoparticles on borosilicate glass substrate, and reference glass substrate. (b) Normalized absorption spectrum of the sample. Inset: The Au nanoparticle deposited area on a glass substrate receiver. For Raman measurements, a 785 nm laser wavelength is used. This represents the spectral band indicated.

### 3.2 Raman Measurements from Samples

An initial experiment is performed on a silicon wafer (University Wafer, 5 Ω-cm, P-type, boron doped) with ∼1 nm thick native oxide layer.^32^ Au nanoparticles are deposited on top of the wafer, and the Raman and SERS measurements, as shown in Figure 7, are respectively performed outside and inside the nanoparticle deposition area. It is observed that the deposition of Au nanoparticles enhances Raman features associated with the native SiO_*x*_ overlayer^33^ on the silicon wafer. In addition to the sharp crystalline-Si mode at ∼520 cm^–1^, the SERS spectrum exhibits broad features in the ∼300–400 cm^–1^ region and a distinct band near ∼800 cm^–1^. These bands are consistent with vibrational modes of silica/silicon-oxide networks, for which the 400–600 cm^–1^ region is commonly assigned to Si–O–Si bending and the ∼800 cm^–1^ band to symmetric bridging-oxygen, O–Si–O-related motion.^34,35^ Because the ∼400 cm^–1^ region can overlap with broader amorphous-oxide and weaker silicon-related contributions, these bands are best interpreted as enhanced native-oxide features superimposed on the silicon background.^36^ This interpretation is further supported by prior studies comparing Si with native oxide and oxide-free Si in plasmonic SERS-related measurements, which discuss that the presence or absence of the native oxide layer modifies the optical response and the observed Raman signature.^37,38^ These results clearly show that ∼1 nm thick native oxide can be detected by the SERS method and support the attempt to detect surface properties of planetary materials. In addition to this, the enhanced background that reduces with increasing wavenumber in the 200–500 cm^–1^ range could be attributed to inelastic light scattering due to Au nanoparticles.^39^ So, in particular, the presence of Au nanoparticles causes the surface enhancement of the ∼1 nm thick layer of the oxide, which was not detected in the conventional Raman spectrum in Figure 7. This shows the ability of SERS to detect the surface layers on materials.

**Figure 7:**
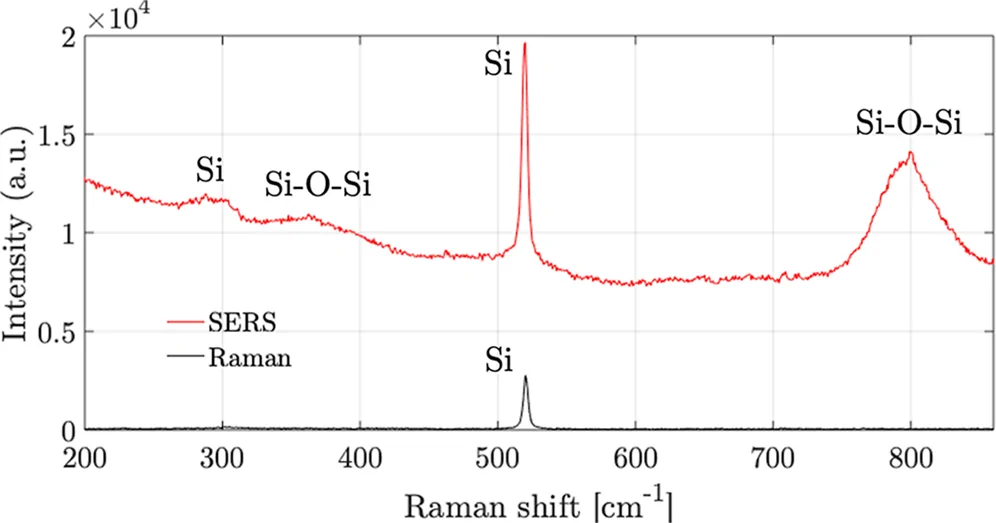
Raman measurements from a silicon wafer.

The measurements on the mineral sample prepared in Section 2.4 are described next. The surface images of the sample (simulants on the olivine substrate described in Figure 2) are shown in Figure 8. These images of each of the simulant and the mixture sample are obtained using the Hirox 8800 optical microscope with 1000× magnification. It could be observed that the simulants are typically composed of grains of a few tens of μm, which emulates the similar conditions of surface materials on rocky bodies. The thickness of each of the thin layers is measured as ≈5.5 μm, by measuring the vertical shift needed to focus the top surface of the simulant layer relative to the surface of the olivine substrate. Thin-layer uniformity was controlled by keeping the dispersion composition, application procedure, and drying temperature constant for all samples. The Raman measurements for the case of CSM-LHT simulant layer are shown in Figure 9a.

**Figure 8:**
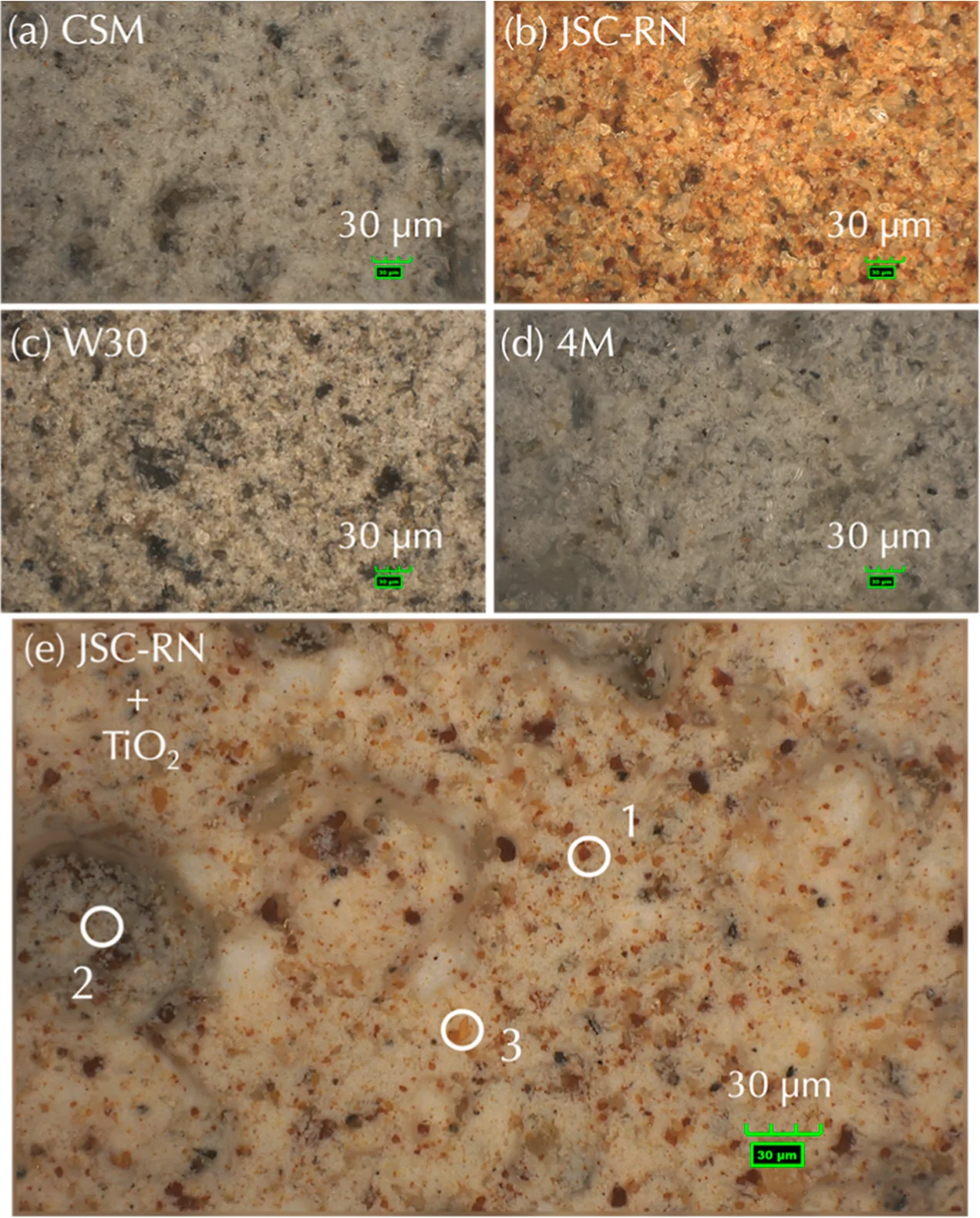
High-resolution optical microscope images of thin films of (a) CSM, (b) JSC-RN, (c) W30, (d) 4 M, (e) JSC-RN and TiO_2_ (anatase) mixture, with three Raman measurement positions indicated as 1, 2, and 3. Scale bar indicates 30 μm.

**Figure 9:**
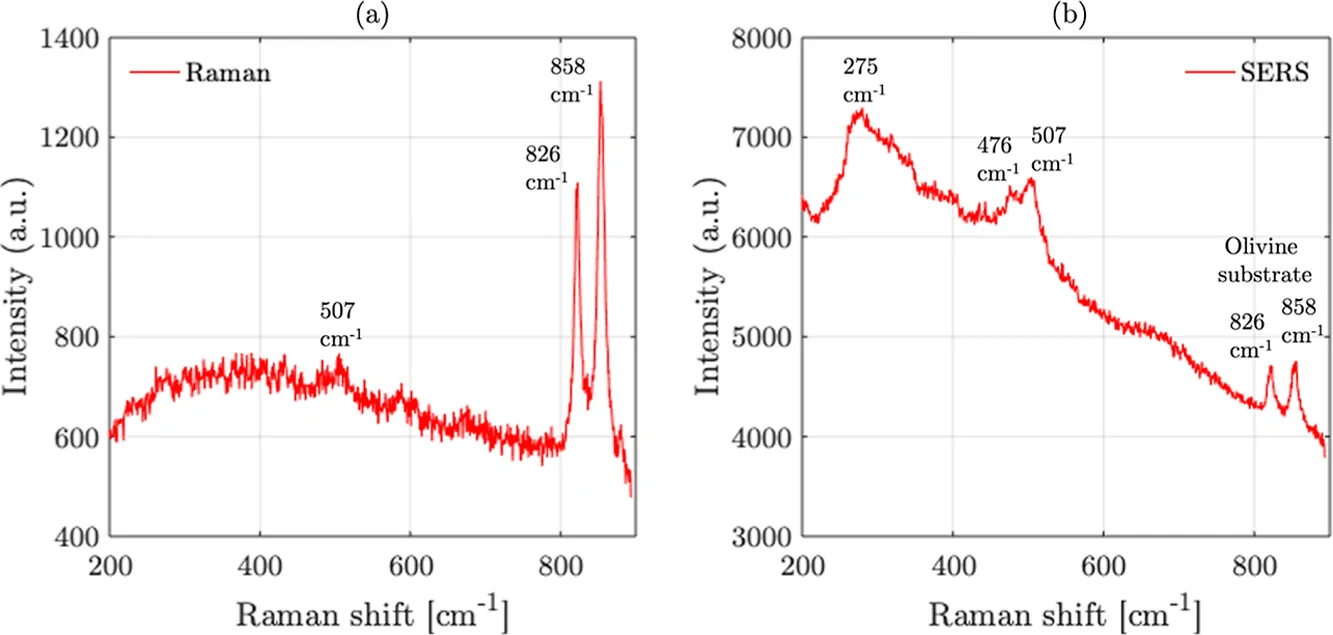
Raman spectra from CSM-LHT simulant on olivine substrate: (a) without nanoparticles, (b) with Au nanoparticles.

Two prominent twin Raman peaks at 826 cm^–1^ and 858 cm^–1^ can be observed, which correspond to the Raman scattering from the bulk olivine substrate.^40,41^ Significantly weaker signatures ∼500 cm^–1^ can also be observed, which correspond to the plagioclase mineral constituent of the CSM-LHT simulant. However, the other constituent features of CSM-LHT cannot be observed in Figure 9a. This stems from the fact that the simulant is a thin layer of ∼20 μm on the top of the olivine mineral. This demonstrates that conventional Raman spectroscopy cannot be used to detect surface-level thin-layered materials using standard spectrometers, which appear as long-term surface alterations or impurities on the planetary materials but contain important planetary information. Nonetheless, when probed over the nanoparticle deposited area, the Raman peaks are significantly enhanced as shown in Figure 9b. The Raman peaks at 476 cm^–1^ and 507 cm^–1^ are more distinct, along with the detection of broad features with a peak at ∼276 cm^–1^. The observations from Figure 9b correspond, respectively, to the constituent composition of CSM-LHT containing the plagioclase mineral^42^ and a mixture of pyroxenes and glass agglutinates,^43^ which are expected materials in the lunar regolith. For comparison, the Raman measurements of CSM-LHT in the bulk form have been reported in a previous publication.^44^ The prominent Raman peaks from olivine at 826 cm^–1^ and 858 cm^–1^ are still visible in Figure 9b. It is to note that while the Raman peaks of CSM-LHT thin surface layer were enhanced, the Raman peaks from the bulk are not, which corresponds to the surface-level influence of the surface plasmons from gold nanoparticles, allowing only the enhancement of the Raman signal from surface-level materials and not from the bulk. The reduction in relative olivine peak intensity after nanoparticle deposition is consistent with the enhanced near-surface response of the simulant layer and the change in the local optical environment introduced by the Au nanoparticles. At the same time, the background increases because the nanoparticle-coated rough powder layer promotes additional scattering from the grains and from the heterogeneous surface topography. As a result, the measured spectrum contains a stronger continuum background even as the surface-layer Raman features become more distinct.

The Raman measurements for the case of JSC-RN simulant layer are shown in Figure 10a.

**Figure 10:**
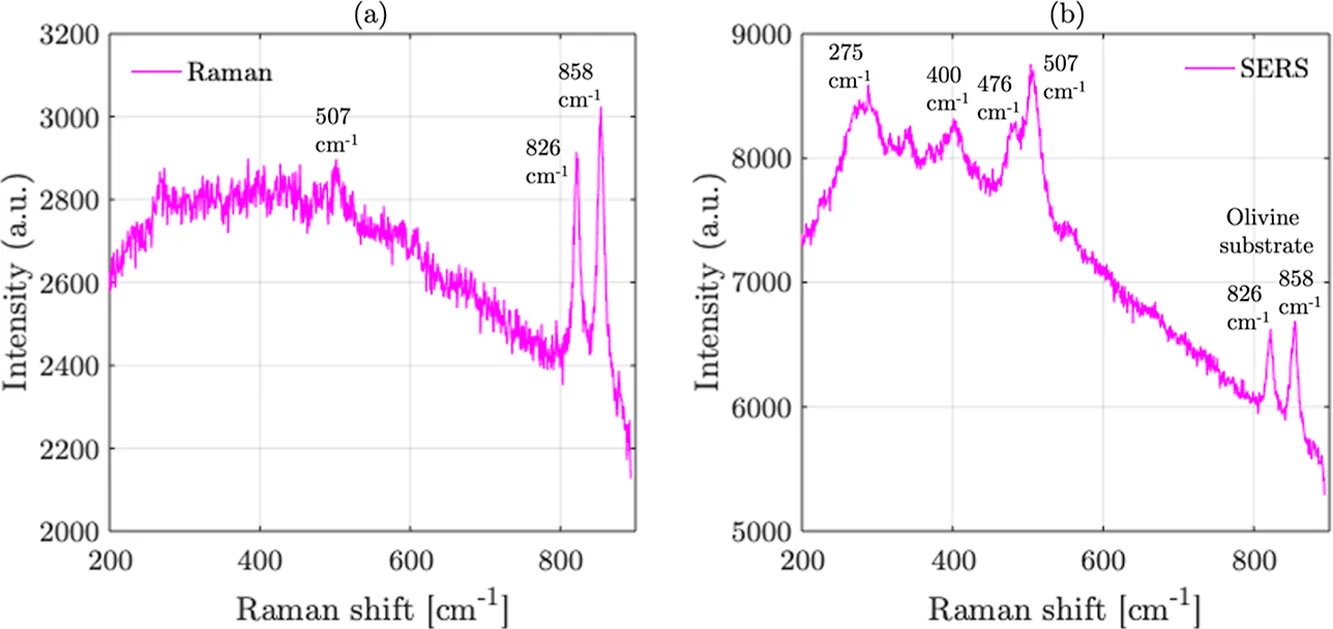
Raman spectra from JSC-RN simulant on olivine substrate: (a) without nanoparticles, (b) with Au nanoparticles.

Similar to the previous example, the constituent twin Raman peaks at 826 cm^–1^ and 858 cm^–1^ can be observed, which correspond to the Raman scattering from the bulk olivine substrate. Significantly weaker signatures at 275 cm^–1^ and ∼500 cm^–1^ can also be observed, which correspond to the plagioclase mineral constituent of the JSC-RN simulant. Weaker features are due to the thinness of the simulant layer compared to the bulk substrate, where significantly more scattering volume is available to produce distinct constituent peaks of olivine from the bulk. However, when measured on the area with deposited nanoparticles, more distinct Raman features of plagioclase^42^ are observed at 275 cm^–1^, 402 cm^–1^, 476 cm^–1^ and 507 cm^–1^, and a weak feature at 555 cm^–1^, that allows to identify the specific plagioclase mineral anorthite as shown in Figure 10b. This results from the surface-level Raman enhancement by virtue of plasmonic Au nanoparticles. The other Raman features with peaks at 344 cm^–1^ and 404 cm^–1^, are also observable, which correspond respectively to the plagioclase altered phases and feldspathic matrix,^45^ constituent of the JSC-RN simulant. For comparison, the Raman measurements of JSC-RN in the bulk form have been reported in a previous publication.^44^ The bulk olivine peaks are also present but not enhanced owing to the surface-level influence of the plasmonic nanoparticles.

The Raman measurements for the case of W30 simulant layer are shown in Figure 11a. The twin Raman peaks at 826 cm^–1^ and 858 cm^–1^ can be observed, which correspond to the Raman scattering from the bulk olivine substrate. While weaker features of plagioclase at ∼500 cm^–1^ are barely discernible in Figure 11a, probing with the Au nanoparticle deposited area results in significantly enhanced Raman features of the surface-level W30 simulant layer, as shown in Figure 11b. Similar to the previous examples, the anorthite plagioclase features at 275 cm^–1^, 400 cm^–1^, 439 cm^–1^, 476 cm^–1^, 507 cm^–1^, and 687 cm^–1^, are observable. Weaker pyroxene peaks at 327 cm^–1^ and 346 cm^–1^ can also be observed. For comparison, the Raman measurements of W30 in the bulk form have been reported in a previous publication.^44^

**Figure 11:**
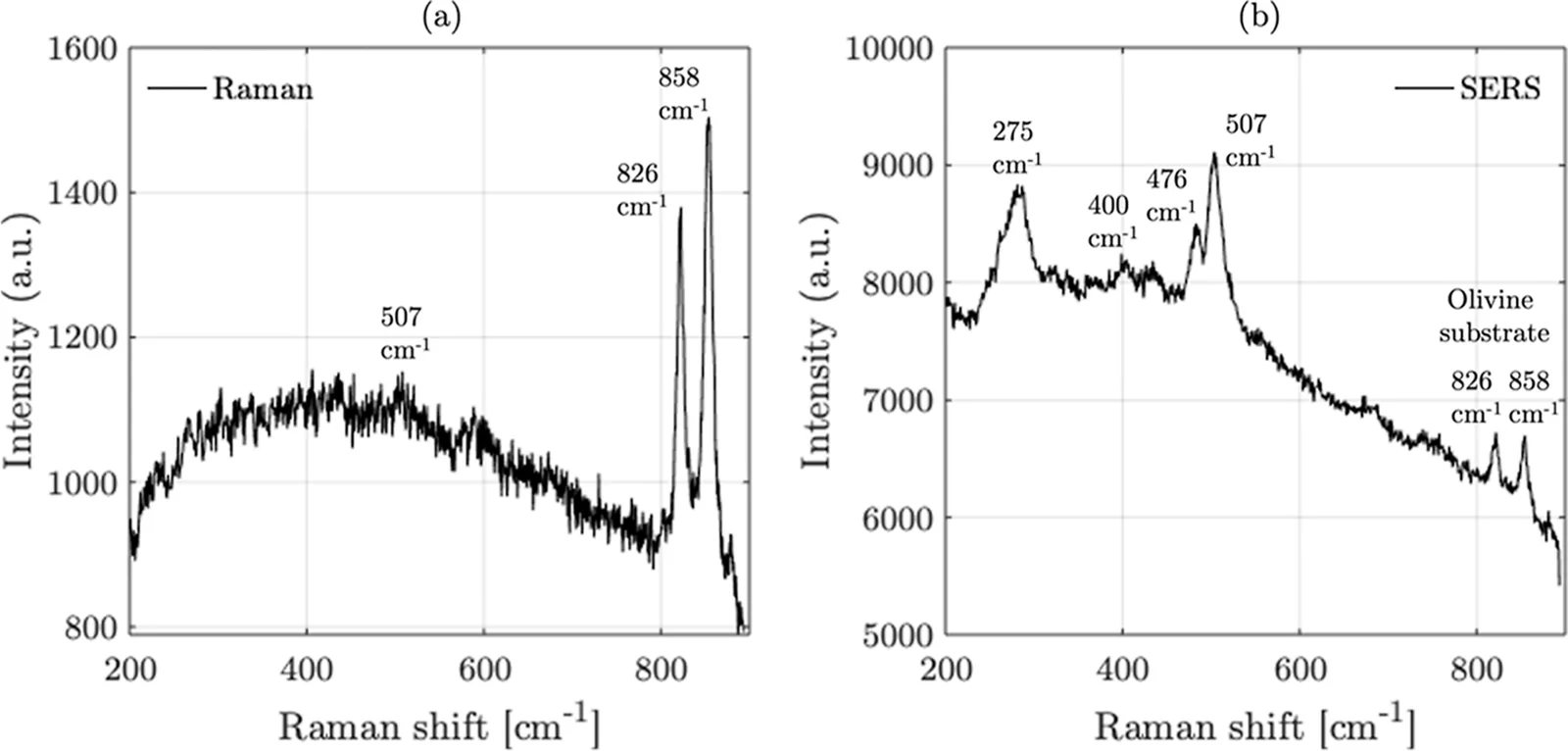
Raman spectra from W30 simulant on olivine substrate: (a) without nanoparticles, (b) with Au nanoparticles.

The Raman measurements for the case of 4 M simulant layer are shown in Figure 12a. The constituent twin Raman peaks at 826 cm^–1^ and 858 cm^–1^ can be observed, which correspond to the Raman scattering from the bulk olivine substrate. Similar to the previous examples, while weaker plagioclase features are observable, probing on the Au nanoparticle area results in significant enhancement in the plagioclase features (Figure 12b) with prominent peaks occurring at 275 cm^–1^, 400 cm^–1^, 476 cm^–1^, 507 cm^–1^, 507 cm^–1^, 559 cm^–1^ and 739 cm^–1^, with pyroxene peaks observable at 324 cm^–1^, 341 cm^–1^ and 676 cm^–1^. The bulk olivine twin peaks are observable at 826 cm^–1^ and 858 cm^–1^. For comparison, the Raman measurements of 4 M in the bulk form have been reported in a previous publication.^44^ In summary, the distinct Raman peaks of anorthosite and pyroxene are observed with the help of surface-level enhancement of Raman scattering from the thin simulant layer, which cannot be detected from the thin-layer using a conventional Raman measurement.

**Figure 12:**
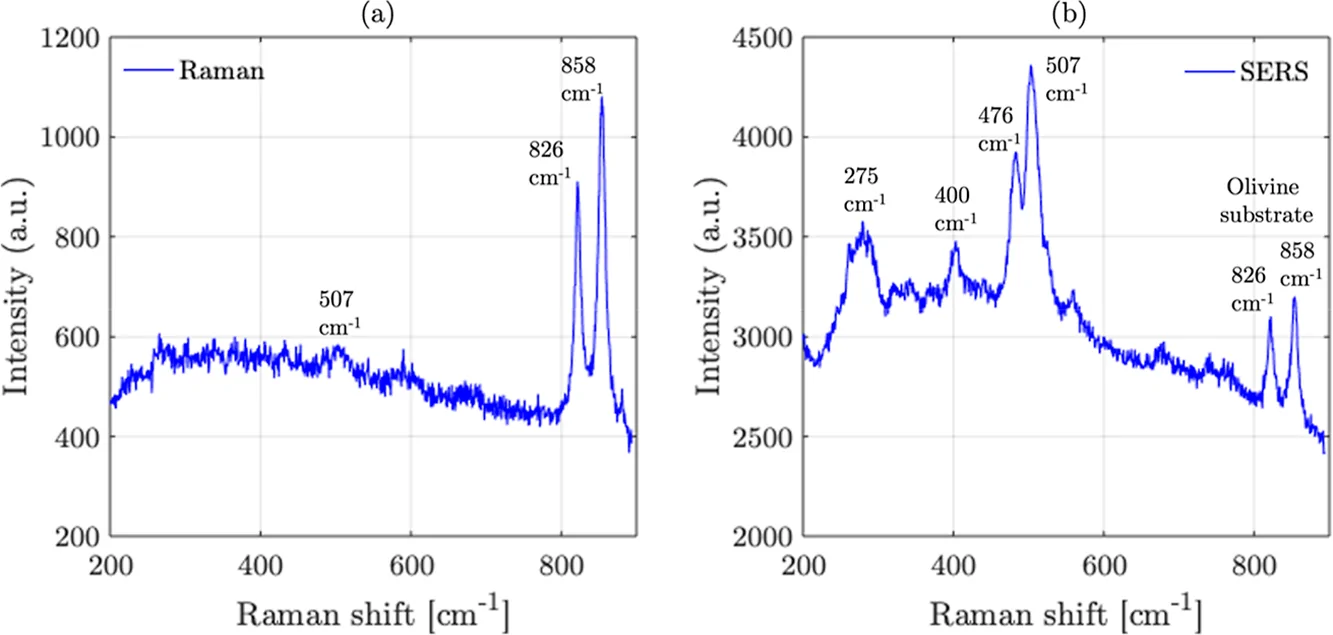
Raman spectra from 4 M simulant on olivine substrate: (a) without nanoparticles, (b) with Au nanoparticles.

As mentioned in Section 2.2, an additional thin-layer sample was made on the olivine substrate as a mixture of two materials, TiO_2_ (anatase) and JSC-RN, with the aim of sensitively detecting each component in the mixture. Three specific locations, 1, 2, and 3 were chosen for the SERS measurements, as shown in Figure 8e. The Au nanoparticles were deposited over this entire image area, covering all three specific locations 1, 2, and 3. The measurements are shown in Figure 13.

**Figure 13:**
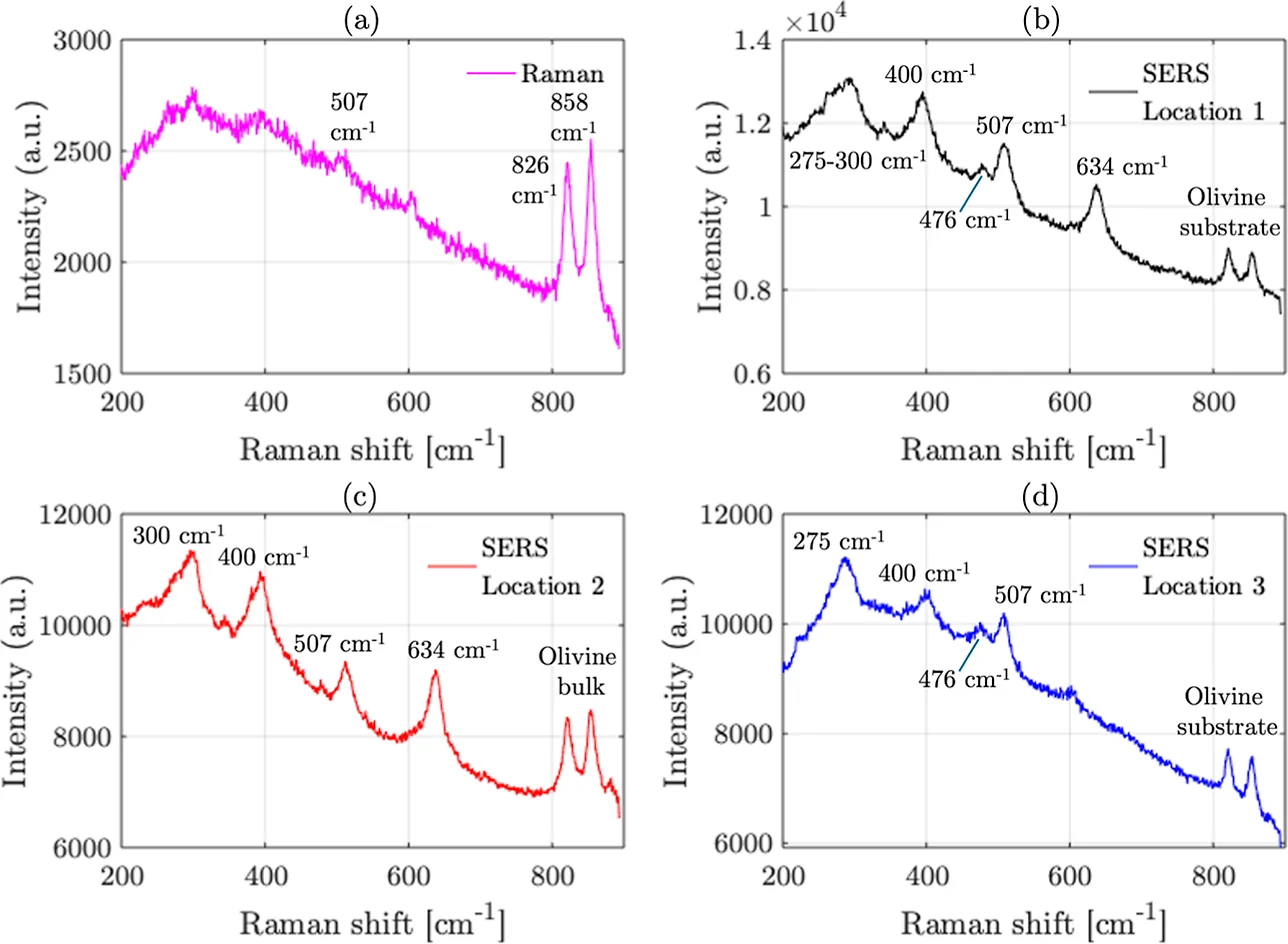
Mixture detection: (a) without nanoparticles. (b–d) with Au nanoparticles at 1, 2, and 3 locations.

When measured in an area without nanoparticles, the Raman measurements are observed as shown in Figure 13a. The twin Raman peaks at 826 cm^–1^ and 858 cm^–1^ are observable, corresponding to the Raman from the bulk olivine substrate. The constituent plagioclase peaks of JSC-RN and the TiO_2_ anatase peak, expected at ∼634 cm^–1^ are weakly detectable. The Raman measurements from TiO_2_ anatase have been reported in a previous publication.^44^ In the case of probing over the nanoparticle area, in location 1, the surface enhanced Raman measurements are shown in Figure 13b. The Raman features of both JSC-RN are observable in the 275–500 cm^–1^ along with the TiO_2_ (anatase) at 400 cm^–1^ or 634 cm^–1^. An additional location, location 2, was probed where the TiO_2_ (anatase) and JSC-RN mixture was similar compared to location 1. As shown in Figure 13c, here too, the constituent Raman peaks are detectable, however, with an enhancement in the olivine twin peaks as well. This owes to the fact that dark-colored olivine substrate is exposed to the Au nanoparticles more in the vicinity of location 2, which allows the olivine to be possibly in contact with Au nanoparticles. Furthermore, to specifically detect JSC-RN grains, an additional location 3 was identified. As shown in Figure 13d, the measurements in this area resulted in the enhancement of Raman peaks specific to the JSC-RN simulant, and the weakly observable TiO_2_ (anatase) peak at 400 cm^–1^ and 634 cm^–1^. These mixture detection measurements highlight the capability of the laser deposition technique to detect 2- or 3- phase systems as well (3- phase because the olivine twin peaks are also detectable from the bulk).

To summarize the measurement set and the comparison experiments reported in this work, Table 2 lists the sample types and primary outcomes.

**Table 2:** A Summary of the Measurements Performed

sample for measurement	purpose	SERS main observation
silicon wafer with native oxide	verify surface sensitivity on an ultrathin silicon oxide layer	enhanced SiO_*x*_-related Raman features from the native oxide layer
CSM-LHT on olivine	detect thin lunar analog surface layer	plagioclase-related peaks become distinct after nanoparticle deposition
JSC-RN on olivine	detect Martian analog surface layer	enhanced plagioclase and feldspathic matrix features
OPRH4-W30 on olivine	detect lunar analog surface layer	enhanced surface-layer response with weak bulk contribution
NU-LHT-4 M on olivine	detect lunar analog surface layer	enhanced plagioclase features
Mixture of JSC-RN and TiO_2_ on olivine	detect multiphase surface system	raman signatures from both components are detected

### 3.3 A Comparison of Laser Deposition of Nanoparticles with a Liquid Colloidal Solution Method

For comparison with liquid colloidal solution, SERS measurements were also performed using commercially available Ag nanoparticles (mean diameter of 65 nm) deposited from a liquid colloidal solution (0.02 mg/mL Ag nanoparticles in 2 mM sodium citrate aqueous buffer, Sigma-Aldrich) onto the olivine surface and probed with a 532 nm excitation wavelength. The SEM image with deposited liquid solution nanoparticles is shown in Figure 14a, and the resulting spectra are shown in Figure 14b. In comparison with Figure 4a for Au nanoparticles, the SEM image of Ag nanoparticles shows the presence of nanoparticle clusters nonuniformly covered over the area. An enhancement of the olivine Raman peaks at 826 cm^–1^ and 858 cm^–1^ is observed (∼10^4^) after nanoparticle deposition. Similar results would be expected for gold nanoparticles. The Ag nanoparticles were used as they were available in the laboratory.

**Figure 14:**
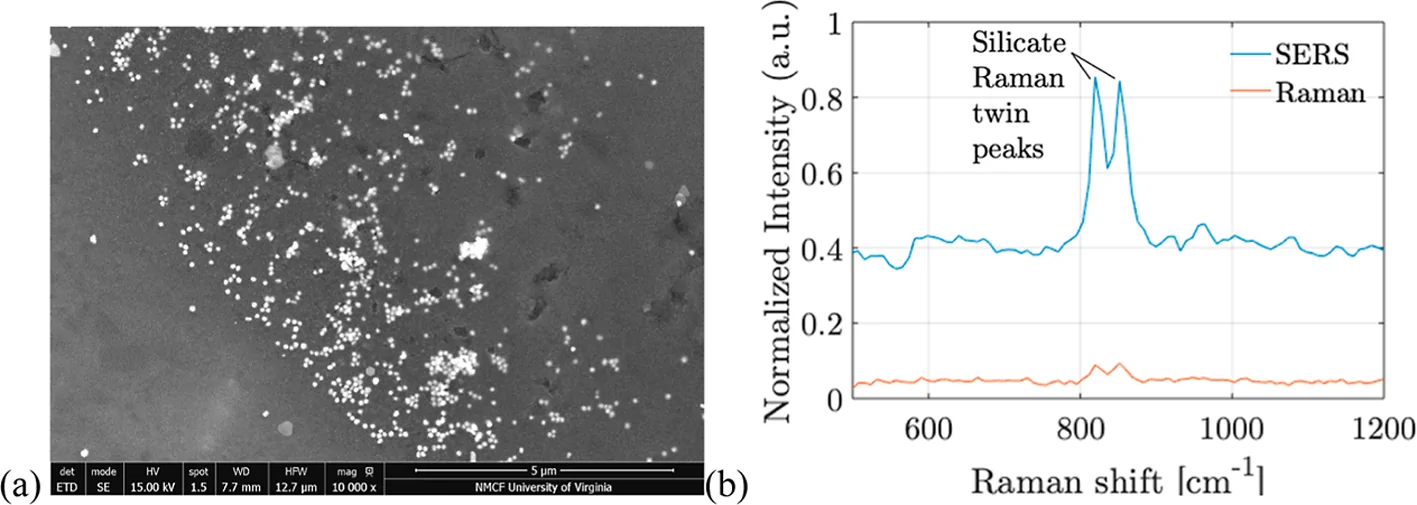
(a) SEM image of Ag nanoparticles, (b) and measurements of SERS on an olivine sample with nanoparticles deposited using a liquid colloidal solution.

## 4 Discussion

The laser deposition of Au plasmonic nanoparticles was used to enhance the surface-sensitive detection of thin material layers on a planetary material. The overall objective was to show that this approach can enhance the Raman response of planetary surface materials without requiring liquid colloids or conventional prefabricated SERS substrates commensurate with in situ techniques viable in a resource limited robotic mission or astronaut fieldwork. The presence of colloids can complicate planetary protection requirements. In comparison, laser-deposited Au nanoparticles are dry, inert, low reactive, low catalytic, and minimally toxic.^21,22^ Gold is highly resistant to oxidation and is widely recognized for its chemical stability and biocompatibility, minimizing unwanted chemical contamination of the planetary sample surface while preserving the plasmonic sensing capability. Further, by selectively depositing nanoparticles only on the region of interest, the technique enables localized, in situ interrogation of surface-level materials while preserving the surrounding areas of the sample.

A major scientific motivation for surface-sensitive measurements is that the most diagnostically important material on planetary bodies is often concentrated in the uppermost few tens of microns of the surface. Dust coatings, weathering products, irradiation-induced changes, and thin alteration films can all carry information about surface history, environmental exposure, and local chemistry.^46–49^ Because these surface layers may be extremely thin relative to the penetration depth of a Raman probe, a method that selectively amplifies the near-surface response is needed to isolate their signatures from the much stronger signal of the underlying substrate. This work has successfully demonstrated a method to probe surfaces of solid materials without the need for a liquid solution.

Compared with prior laser-based SERS studies, the present work introduces a new sensing configuration and application domain. Earlier laser-method studies have primarily focused on fabricating SERS-active substrates, nanoparticle patterns, or aggregated plasmonic surfaces for later detection of separate analytes.^18–25^ In contrast, this work uses laser-generated Au nanoparticles directly on a rough planetary-relevant mineral substrate to interrogate thin surface layers in situ, without relying on a prefabricated SERS substrate or a liquid colloidal coating. The method, therefore, combines dry nanoparticle generation, localized deposition, and surface-selective Raman enhancement in a way that is particularly well suited to heterogeneous planetary materials. Beyond planetary samples, the dry and localized deposition approach may be valuable for geological applications on Earth, where rough, heterogeneous surfaces and thin alteration films often complicate conventional Raman measurements. The presented method is well-suited for the analysis of a variety of solid surfaces.

The silicon wafer experiment initially confirms the surface sensitivity of the method. The appearance of enhanced features from the ∼1 nm native oxide layer demonstrates that laser-deposited plasmonic nanoparticles can probe extremely thin surface films, providing confidence in the detection of the much thicker simulant surface layers on olivine.

In the case of simulants, across all the simulants cases studied, the key Raman features associated with the plagioclase-rich surface layers became significantly more distinct after Au nanoparticle deposition. In the absence of nanoparticles, the thin simulant layers produced only weak signatures because the simulant thickness was small compared with the optically active volume of the underlying olivine substrate. Once the plasmonic nanoparticles were introduced, however, the surface-layer plagioclase Raman bands at 275 cm^–1^, 400 cm^–1^, 496 cm^–1^, and 507 cm^–1^ became more distinct while the dominant olivine peaks at 826 cm^–1^ and 858 cm^–1^ remained visible but were not comparably enhanced. This behavior is consistent with the near-surface nature of the plasmonic field, which preferentially amplifies Raman scattering from materials in direct proximity to the nanoparticles. The observations therefore support the central premise of the work, that is, the laser-deposited plasmonic nanoparticles can selectively enhance the spectral response of thin solid surface deposits that would otherwise be difficult to detect by conventional Raman spectroscopy.

The measured Raman signal enhancement is particularly important from the perspective of planetary science, where thin surface coatings, dust mantles, weathered layers, and other near-surface alterations may contain critical mineralogical or environmental information. On Mars and the Moon, such surface-level materials may accumulate over long time scales, and they may differ from the underlying bulk material in composition, crystallinity, or alteration state. The present results suggest that SERS-based detection using laser-deposited gold nanoparticles can access such near-surface information while still retaining the bulk mineral Raman signatures. This dual response is advantageous because it provides simultaneous access to both the surface deposit and the underlying substrate, thereby offering a more complete picture of the sample microstructure and composition. Particularly, both normal Raman and SERS measurements can be obtained from planetary surfaces just as were obtained from the samples in this work, which helps identify strictly surface species.

The estimated enhancement factor further supports the usefulness of the approach. Previous studies calculate the enhancement factor by multiplying the amplification in the intensity of the Raman spectrum by the ratio of the penetration depth of the laser beam into the sample to the near-field depth of plasmonic influence.^50,51^ In the present measurements, the plagioclase-related Raman signal increased by roughly an order of 10× when comparing spectra with and without nanoparticle deposition. When this enhancement is scaled by the ratio of approximate thickness (∼5.5 μm) of the simulant layer to the nanometric region of plasmonic influence (assuming ∼10 nm, which is a typical value for near-field plasmonic penetration depth^52^), the effective enhancement reaches ∼10^4^. The depth of penetration of a 785 nm wavelength is in the order of a few μm in mineral samples,^53^ which is the reason why the thickness of the thin layer of the simulant is considered in the calculations instead of the entire penetration depth of the laser beam in the simulant sample. This level of enhancement in the order of 10^4^ from the top surface of the simulants is sufficient to make weak surface-layer signals clearly distinguishable above the background, amid the strong Raman response of the underlying bulk olivine. At the same time, the choice of a 785 nm excitation wavelength helps minimize fluorescence contributions that are often problematic at shorter visible wavelengths, thereby improving the overall spectral clarity for planetary materials sensing applications.

The estimated enhancement of ∼10^4^ should be viewed as a conservative effective enhancement rather than a theoretical maximum. Several factors limit the measured value, including the broad particle size distribution of the deposited Au nanoparticles, the nonideal resonance matching between the plasmon peak and the 785 nm excitation wavelength, the rough and heterogeneous nature of the surface layer, and the fact that the reported enhancement is derived from a thin-layer-to-nanometric-interaction-depth scaling. In addition, the experiments were performed under ambient conditions, which can broaden the nanoparticle distribution and reduce the uniformity of the near-field enhancement. Even with these constraints, the enhancement is sufficient to reveal weak surface-layer Raman bands that are otherwise hidden by the substrate response. It is to mention that the enhancement estimate was obtained from multiple spectra collected at representative nanoparticle-deposited locations on the simulant surfaces. The reported value reflects a representative surface-enhancement trend rather than a single isolated hotspot. Measurements at multiple positions showed the same qualitative enhancement behavior, with the plagioclase bands consistently becoming more distinct after Au nanoparticle deposition. A full statistical treatment of enhancement across all positions is limited by the heterogeneous nature of the rough simulant surfaces. So, the value reported here should be interpreted as an effective average enhancement for the present deposition conditions.

The particle statistics and optical resonance also provide useful insight into the performance of the deposition process. The deposited Au nanoparticles showed a broad size distribution under ambient conditions, with the mean size decreasing as laser power increased. The measured plasmonic resonance peak around 720 nm wavelength is reasonably well matched to the 785 nm Raman excitation wavelength, supporting the observed SERS response. At the same time, the data suggests further optimization could be realized. Better control of nanoparticle shape and size distribution, for example, through modified deposition conditions or the use of anisotropic particle geometries such as nanorods, nanostars, or nanofractals, could improve the resonance matching and increase the enhancement efficiency.^54–57^ Similarly, the use of alternative plasmonic materials such as silver could shift the resonance further into the visible range, depending on the target excitation wavelength and application needs.^58–60^

The mixture experiments additionally demonstrate that the technique is not limited to single-component surface layers. The Raman signatures of the constituent mineral of JSC-RN and the Raman signature of TiO_2_ (anatase) could be detected when nanoparticles were deposited over the mixed surface region, confirming that the method can be extended to multiphase systems. In some locations, the olivine contribution became more pronounced, reflecting variations in local coverage, surface exposure, and the spatial distribution of the simulant mixture. This local variability is realistic for natural planetary material surfaces and highlights the importance of region-specific probing. The results, therefore, indicate that the laser deposition approach is suitable not only for detecting isolated surface deposits but also for identifying coexisting phases in heterogeneous planetary materials.

Importantly, the present experiments were carried out under ambient laboratory conditions, which reinforces the practical simplicity of the method. Although the current nanoparticle size distribution is relatively broad, the approach already provides clear spectral enhancement. Under more controlled environments, such as vacuum or microgravity as encountered on planets, the deposition process may yield narrower particle distributions and better SERS enhancement. Even in its present form, however, the technique demonstrates that localized laser deposition of plasmonic nanoparticles can serve as an effective route for in situ Raman sensing of surface-level materials or changes to the planetary materials.

For comparison with commonly used liquid colloid deposited nanoparticles, a separate SERS measurement was also performed using 100 nm Ag nanoparticles deposited from a liquid colloidal solution onto olivine and probed with 532 nm excitation. The observed enhancement was on the order of 10^4^, which is comparable to the enhancement obtained here with laser-deposited Au nanoparticles. This comparison indicates that the dry laser-deposition approach can achieve SERS performance similar to the conventional alternative method of liquid-colloid deposition while offering important practical advantages for planetary measurements. In particular, liquid colloids spread over rough mineral surfaces, produce nonuniform coverage, and leave particle agglomeration near the evaporation rim, whereas laser deposition generates and transfers nanoparticles directly onto the selected region without wetting the surrounding surface. This makes the laser method especially suitable for rough, heterogeneous planetary materials, where localized and contamination-free surface deposition is required.

In the future, because many planetary targets of interest occur as thin deposits or surface-bound films, this approach may also be relevant to the detection of trace hydrated phases, water-bearing materials, and ice-related surface deposits on cold planetary environments. Also, the same strategy could be extended to surfaces where volatile or weakly scattering phases are present only as near-surface coatings or mixed grains. This is also applicable for the detection of phyllosilicates.^61^ Furthermore, although isotopic composition is not directly measured in the present study, the method may be relevant to isotope-related planetary investigations as an enabling surface-analysis tool. In particular, many isotope-sensitive measurements depend on identifying the correct mineral phase, alteration product, or deposited surface material before isotopic characterization can be interpreted correctly. A localized SERS capability could assist in the preliminary identification of such phases, especially when the material of interest exists only as a thin surface layer or mixed deposit on a rough substrate. In that sense, the method may support future phase identification for isotope-focused planetary studies. In addition to this, although the present work is motivated by planetary exploration, the same approach may also be useful for Earth-based geological sensing. Thin alteration layers, weathering films, mineral coatings, and sparse surface contaminants are common in terrestrial rocks, sediments, and industrial mineral samples, and they can be difficult to detect without a surface-selective enhancement mechanism. The ability to deposit nanoparticles locally and probe only a chosen region could therefore be useful in geological field studies, laboratory mineralogy, and the examination of fragile or heterogeneous surface materials on Earth as well. The method is applicable for the analysis of a variety of solid surfaces.

## 5 Conclusions

This work demonstrates the feasibility of using laser deposition of Au plasmonic nanoparticles as a simple and effective route for surface-enhanced Raman spectroscopy of thin surface-level materials on planetary materials surfaces. More broadly, the method may also support geological surface analysis on Earth, especially for thin coatings, weathering products, and heterogeneous mineral surfaces.

Initially, the silicon wafer result shows that the method is capable of detecting about ∼1 nm surface oxide layers with the appearance of Si–O–Si Raman mode in the SERS spectrum. This reinforces its suitability for surface-sensitive Raman probing of thin deposits on planetary-relevant materials. Thereafter, using olivine as a relevant base material and several lunar and Martian simulants (CSM-LHT, JSC-RN, OPRH4-W30, NU-LHT-4M) as thin ∼20 μm surface layers, the experiments show that Raman features that are only weakly observable, or completely undetectable, by conventional Raman measurements become distinctly measurable after Au nanoparticle deposition. The enhanced detection of plagioclase-rich simulant layers, as well as the successful identification of multiphase mixtures such as JSC-RN and TiO_2_ (anatase) confirms that the technique is capable of sensing both single-phase and composite surface deposits. An enhancement factor of ∼10^4^ times was achieved. Importantly, this enhancement remains localized to the surface region in the top nanolayers, while the underlying olivine Raman signatures remain observable, supporting the near-surface selectivity of the plasmonic effect.

The results also show that the laser deposition process offers practical flexibility in controlling nanoparticle size and resonance behavior, with plasmonic response tuned toward the 785 nm Raman excitation wavelength. This could lead to the development of new SERS instruments in conjunction with the current Raman systems.^62–66^ Although the current experiments were performed under ambient conditions and produced a broad nanoparticle size distribution, the technique already yields clear SERS enhancement and strong spectral contrast for thin surface layers. Future improvements may include better control of nanoparticle geometry, use of alternative plasmonic metals, and deployment under vacuum or microgravity conditions to improve uniformity and enhancement efficiency, including responses in cryogenic conditions.

## Data Availability

All data is available presented throughout the manuscript.
